# Fabrication of Polyvinylidene Difluoride Membrane with Enhanced Pore and Filtration Properties by Using Tannic Acid as an Additive

**DOI:** 10.3390/polym14010186

**Published:** 2022-01-03

**Authors:** Sri Mulyati, Sri Aprilia, Syawaliah Muchtar, Yanna Syamsuddin, Cut Meurah Rosnelly, Muhammad Roil Bilad, Shafirah Samsuri, Noor Maizura Ismail

**Affiliations:** 1 Department of Chemical Engineering, Universitas Syiah Kuala, Banda Aceh 23111, Indonesia; sriaprilia@unsyiah.ac.id (S.A.); syawaliah@unsyiah.ac.id (S.M.); yanna_syamsuddin@unsyiah.ac.id (Y.S.); cut.meurah@che.unsyiah.ac.id (C.M.R.); 2 Faculty of Integrated Technologies, Universiti Brunei Darussalam, Gadong BE1410, Brunei; 3 Chemical Engineering Department, Universiti Teknologi PETRONAS, Seri Iskandar 32610, Perak, Malaysia; shafirah.samsuri@utp.edu.my; 4 Faculty of Engineering, Universiti Malaysia Sabah, Kota Kinabalu 88400, Sabah, Malaysia; maizura@ums.edu.my

**Keywords:** tannic acid, ultrafiltration, humic acid, antifouling

## Abstract

Potential use of tannic acid (TA) as an additive for fabrication of polyvinylidene difluoride (PVDF) membrane was investigated. The TA was introduced by blending into the dope solution with varying concentrations of 0, 1, 1.5, and 2 wt%. The prepared membranes were characterized and evaluated for filtration of humic acid (HA) solution. The stability of the membrane under harsh treatment was also evaluated by one-week exposure to acid and alkaline conditions. The results show that TA loadings enhanced the resulting membrane properties. It increased the bulk porosity, water uptake, and hydrophilicity, which translated into improved clean water flux from 15.4 L/m^2^.h for the pristine PVDF membrane up to 3.3× for the TA-modified membranes with the 2 wt% TA loading. The flux recovery ratio (FRR) of the TA-modified membranes (FRRs = 78–83%) was higher than the pristine one (FRR = 58.54%), with suitable chemical stability too. The improved antifouling property for the TA-modified membranes was attributed to their enhanced hydrophilicity thanks to improved morphology and residual TA in the membrane matric.

## 1. Introduction

Membrane technology has been widely applied for water and wastewater treatments. It offers advantages such as no change of phase, minimum use of chemicals, ease of operation, high selectivity, and relatively low energy consumption [1,2,3]. It has been increasingly popular and is widely applied in water and wastewater treatments, such as for heavy metal removal [4], hospital waste [5], removal of organic compounds such as humic acid (HA) [6], and many others.

Polyvinylidene difluoride (PVDF) is one of the most popular materials for membrane fabrication. It offers excellent thermal stability, chemical resistance, and mechanical strength. PVDF is also stable against corrosive chemicals and organic compounds, including acids and oxidants [7]. However, this polymer cannot be used in pure conditions for water purification due to its high hydrophobic and lipophilic properties. One of the methods applied to increase PVDF membranes’ hydrophilicity is by adding hydrophilic additives as modifying agents [8] that improve the structure and surface chemistry of the resulting membranes. Commonly used additives to increase the hydrophilicity of the membrane are polyethylene glycol (PEG) [9], brij [10], pluronic [11], polydopamine [12,13], and others. However, these commercial chemicals are generally quite expensive, which could inflate the price and lower the economic viability of the membranes.

Recently, tannin, a compound from a natural source, was reported as a potential additive for membrane fabrication [14]. Tannin is a type of plant polyphenol with a high content of phenolic hydroxyl groups. Tannin in the form of tannic acid (TA) has attracted great attention of membrane scientists due to its fantastic properties. It is low cost, non-toxicity, hydrophilic nature, and easy processing [15,16]. It has been applied mainly in three aspects: membrane surface modification, interlayers, and selective layers construction, and mixed matrix membrane development [14]. Membrane surface modification by tannic acid offers the following advantages: extremely simple, fast, and applicable to almost any polymeric membrane [14].

TA adheres robustly onto various substrates, including organic and inorganic ones, hydrophilic and hydrophobic ones, with various shapes. It interacts via hydrophobic interaction thanks to its hydrophobic regions [17] onto hydrophobic regions of the polymer matric. It also has abundant phenolic hydroxyl groups that allow interaction via the hydrogen bond [18]. Therefore, it is very attractive to modify the membrane surface via the coating method. It can also form non-covalent and/or covalent interactions with materials [14]. This way, it can be used as a bridge to introduce other functional materials or used as a co-component via co-deposition.

Improving surface hydrophilicity is an effective strategy for increasing water permeability. The hydrophilic surface offers the formation of a hydration layer at the membrane/water interface [19] that aids in repelling foulant, which is usually hydrophobic (such as oil, humic acid, and protein) [20]. TA with its abundant phenolic hydroxyl groups is hydrophilic and can be directly used to improve membrane surface hydrophilicity. Xu et al. [21] coated the poly(vinylidene fluoride) (PVDF) membranes with TA via dip-coating for improving the surface hydrophilicity. Li et al. [22] employed an inkjet printing method for TA coating on the PVDF membrane surface. Due to the improvement of membrane hydrophilicity imposed by the TA layer, the membranes exhibited higher water fluxes. In a recent comprehensive review [14], many studies employed TA with co-component and used it as an anchor that fixed the modification layer on the membrane surface (i.e., TA with polyvinylpyrrolidone (PVP) assembled with Fe^3+^ [23]).

Recently, TA extracted from date palms was used to modify nanofiltration membranes through interfacial polymerization techniques [24]. The results showed that it succeeded in increasing the resulting membrane chemical resistance. In another report, the modification of membrane with tannin through self-assembly technique obtained a membrane with excellent hydrophilicity with a water contact angle reaching <30° [25]. However, the permeation performance of the tannin-modified membranes was still not satisfactory. Although TA has been studied for membrane modifying agents, most of the reports employed either using surface deposition (coating) method, combining tannin with other chemicals, or for fabrication of non-ultrafiltration membranes [26,27,28,29]. To our best knowledge, applications of TA were vastly used for post-treatment and not in situ as an additive during membrane fabrication, which was applied in this study.

This study explored a simple method of developing a PVDF-based ultrafiltration membrane by using TA as an additive via simple blending in the dope solution. Firstly, PVDF membranes were prepared under various loadings of TA. Next, the effects of TA addition on the resulting membrane properties were evaluated. Subsequently, the filtration performances of the membranes were assessed for filtration of HA solution. Finally, the stability of the TA-loaded PVDF membranes under acidic and alkaline treatments was also evaluated.

## 2. Materials and Methods

### 2.1. Materials

PVDF (average Mw ~534,000 by GPC, Sigma-Aldrich, St. Louis, MO, USA) was used as the polymer and dimethylacetamide (DMAC, Sigma-Aldrich, St. Louis, MO, USA) as solvent. TA (Sigma-Aldrich, St. Louis, MO, USA) was used as a sole additive loaded in the dope solutions. HA (Sigma-Aldrich, St. Louis, MO, USA) was used as a model foulant in the filtration process. Distillate water was used as a nonsolvent in the membrane preparation process, feed for the filtration process, and other uses such as solvent and membrane storing. Hydrochloric acid (HCl, Merck, Darmstadt, Germany) and sodium hydroxide (NaOH, Merck, Darmstadt, Germany) solutions were used for evaluating the membrane chemical stability. All the chemicals were used as received without purification.

### 2.2. Membrane Preparation

Four membranes were prepared with various dope solution compositions listed in Table 1. The membranes were prepared by the nonsolvent-induced phase inversion technique. The dope solutions were prepared by first dissolving 15 wt% PVDF in DMAc solvent. TA additive with concentrations of 1, 1.5, and 2 wt% was also added to the dope solution. After degassing, the homogeneous dope solution was cast into a film on a glass plate using a casting knife with a wet thickness of 300 µm. Next, the cast film was immersed into a coagulation bath containing nonsolvent (distillate water) to allow the phase inversion. The film was let idle in the batch until the solid sheet of polymer matric (membrane) was formed and floated to the surface. The ready membrane was then dried at a room temperature of 25 °C.

### 2.3. Membrane Characterization

#### 2.3.1. Surface Hydrophilicity

Membrane surface hydrophilicity was evaluated by measuring the water contact angle. Before measurement, membrane samples were frozen overnight in a freeze-dryer (FD-1000, Eyela, Tokyo, Japan). For each measurement, 1 µL of distillate water was dropped onto the membrane surface. The contact angles between the water microdroplet and the membrane surface were recorded with a contact angle meter instrument (Drop Master 300, Kyowa Interface Science Co., Saitama, Japan). The measurements were performed at 10 points and repeated three times for each membrane sample.

#### 2.3.2. Morphology

The surface morphology of each membrane was observed through scanning electron microscopy (SEM) imaging to study the impact of various TA concentrations. The membrane surface morphology was observed using a Field-Emission Scanning Electron Microscopy (FE-SEM, JSF-7500F, Jeol Co., Ltd., Tokyo, Japan). Before the analysis, samples were freeze-dried (FD-1000, Eyela, Tokyo, Japan) overnight. The membrane sample was coated with an osmium coater (Neoc-STB, Meiwafosis Co., Ltd., Shinjuku, Japan) to form a conductive ultra-thin osmium layer on the sample to impose conductive property.

#### 2.3.3. Surface Chemistry

Fourier transform infrared analysis (FTIR, PerkinELmer Inc., Waltham, MA, USA) was used to identify the functional groups near the top of the membrane matric. The working principle of FTIR is to measure the absorption of infrared radiation at various wavelengths. The infrared (IR) spectrum was measured in the wavenumber range of 400–4000 cm^−1^.

#### 2.3.4. Porosity, Pore Size, and Water Uptake

Membrane bulk porosity was estimated gravimetrically. The wet membrane after the phase inversion was cut, and the surfaces were wiped with tissue paper then weighted. The sample was then dried in an oven with a temperature of 60 °C until a constant membrane weight was reached. The weight data were then inputted into Equation (1) to obtain the porosity. Water uptake analysis was carried out by immersing the membrane in water for a specific time until the membrane weight was constant. The water uptake parameter was then obtained using Equation (2). The Guerout–Elford–Ferry equation (Equation (3)) was used to determine membrane mean pore radius $r_{m}$ (nm) based on the clean water permeability and the porosity data.

(1) $$\varepsilon =\frac{W_{2}-W_{1}}{\rho \;A\;l}\times 100\%$$

(2) $$wu=\frac{W_{2}-W_{1}}{W_{2}}\times 100\%$$

(3) $$r_{m}=\sqrt{\frac{\left(2.9-1.75\varepsilon \right)\;8\;\eta \;l\;Q}{\varepsilon \;A\;\Delta P}}$$

where *W*_2_ (g) is the initial weight of the membrane in wet conditions, meanwhile *W*_1_ (g) represents the weight of the membrane in dry conditions. Meanwhile, *l*, *A*, *ρ*, η, ΔP, and *Q* represent membrane thickness (m), membrane area (cm^2^), water density (0.998 g/cm), water viscosity (8.9 × 10^−4^ Pas), operating pressure (MPa), and volume rate of permeate (m^3^/s), respectively.

#### 2.3.5. Clean Water Permeability

The filtration test was carried out using a lab-made crossflow ultrafiltration set-up with an effective area of 9.6 cm^2^ at an operating feed pressure of 1 bar and a 60 mL/minute feed flow rate. Prior to the experiment, the membrane was first compacted at a pressure of 1 bar until the pure water flux was constant. Following that, the pure water filtration using distillate water as the feed was run for 60 min. The feed water was pumped into the filtration unit using a peristaltic pump (Watson Marlow, Falmouth, UK). The permeate was collected at 10 min intervals until the filtration process reached a stable condition (about one hour). The data collected from this experiment was used to determine the pure water permeability (*L*) using Equation (4).

(4) $$L=\frac{\Delta V}{\Delta t\;A\;\Delta P}$$

where *V* is the volume of permeate (L), *t* is the filtration time (h), *A* is the effective surface area of the membrane (m^2^), and ΔP is the transmembrane pressure (bar).

#### 2.3.6. Filtration of Humic Acid Solution and Antifouling Test

HA solution with a concentration of 50 ppm was used as a model foulant to evaluate the rejection performance and the membrane fouling propensity of the pristine and TA-modified PVDF membranes. The procedure and conditions for the pure water permeability experiment of the HA solution were precisely the same as those applied in the pure water permeability experiment. For rejection of the dissolved HA, the concentration of HA in the feed solution and the filtered permeate were obtained by analysis using UV-Vis spectrophotometry (U-2000, Hitachi Co., Tokyo, Japan) at an absorbance of 280 nm. Equation (5) was then used to calculate the percentage of rejection (R).

The antifouling property was evaluated through the flux recovery ratio (FRR) parameter. After performing the initial pure water filtration, the feed was replaced by 50 ppm HA solution, and the filtration was run until constant flux was obtained. After the HA solution filtration was completed, the membrane was flipped to allow backwashing using distilled water pressure of 1 bar for 10 min for membrane cleaning purposes. After that, the experiment was then continued on the same membrane by changing the feed to distilled water to obtain the second pure water flux. The HA rejection and FRR were calculated using Equations (5) and (6), respectively.

(5) $$R=\frac{C_{feed}-C_{permeate}}{C_{feed}}\times 100$$

(6) $$FRR=\frac{J_{w}}{J_{wr}}\times 100$$

where *C_feed_* (mg/L) is the HA concentration in the feed, *C_permeate_* (mg/L) is the concentration of HA in the permeate, *J_w_* is the initial pure water flux, and *J_wr_* is the second water flux after membrane cleaning.

#### 2.3.7. Chemical Stability

A membrane chemical stability test was intended to determine the stability of the residual tannin in the membrane matric when the membrane was exposed under acidic or alkaline conditions. This test was carried out by immersing the membrane in 1 N HCl solution (pH:1) and 1 N NaOH solution (pH = 14) for seven days. After the immersion was carried out, a pure water filtration test was carried out and compared with the flux on the membrane before immersion.

## 3. Results and Discussion

### 3.1. Membrane Characterization

#### 3.1.1. Surface Hydrophilicity of Membrane

The hydrophilicity of the membrane can be estimated by measuring the water contact angle, which is the tendency or ability of water to wet/adsorb on the membrane surface [30]. The low contact angle indicates that the membrane surface has a high tendency to be wetted by water, or it means that the membrane is hydrophilic [31].

**Figure 1:**
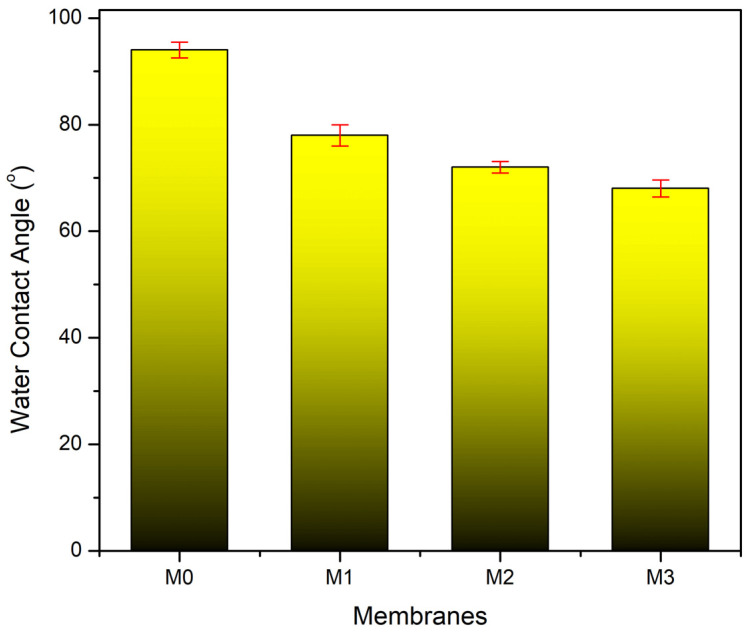
Water contact angle of the pristine PVDF membrane (M_0_) and the ones with the presence of tannic acid at 1 (M_1_), 1.5 (M_2_), and 2 wt% (M_3_) in the dope solutions.

Figure 1 shows the water contact angle of all prepared membranes showing an apparent decrease with increasing loading of TA. The pristine PVDF membrane had the highest contact angle with an average water contact angle value of 94° ± 1.5°. The high water contact angle was attributed to the fluorine content in PVDF that makes this polymer hydrophobic [32]. After adding 1 wt% TA into the dope solution, the contact angle of the M1 membrane reduced to 78° ± 2° and further reduced to 72° ± 1.1° and 68° ± 1.78° with increased concentration of TA to 1.5 and 2 wt%, respectively. The improved hydrophilicity of the membrane with increasing TA concentrations is attributed to the presence of hydrophilic polyphenol in tannin [16,33], which partly resided in the membrane matric. The hydrophilic polyphenol increased the interaction of the membrane surface with water by forming a hydration layer on the membrane wall that provides a hydrogen bonding site for water, resulting in a lower water contact angle [28].

#### 3.1.2. Analysis of Chemical Functional Groups on the Membrane Surface

**Figure 2:**
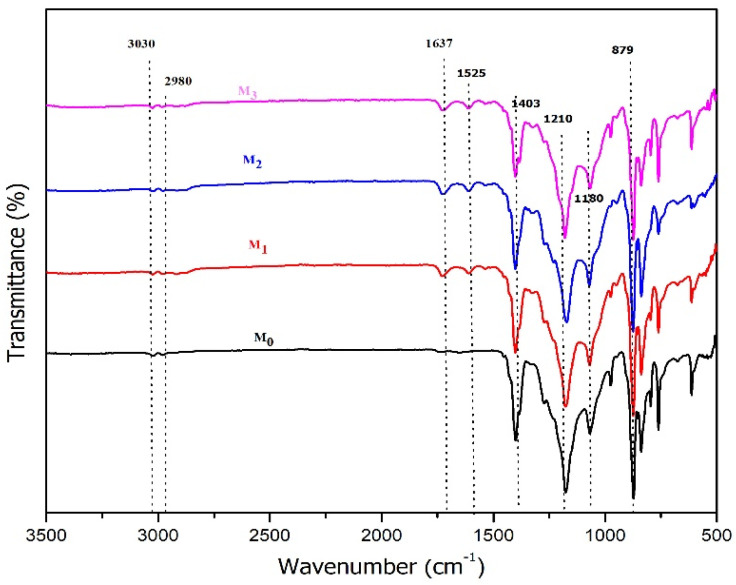
IR spectra of the pristine PVDF membrane (M_0_) and the ones with the presence of tannic acid at 1 (M_1_), 1.5 (M_2_), and 2 wt% (M_3_) in the dope solutions.

The FTIR spectra detailing the chemical functional groups presented on the membrane surface are shown in Figure 2. For the pristine PVDF membrane (M_0_), peaks at the wavelengths of 3030 and 2980 cm^−1^ represented asymmetric and symmetrical vibrations of the C-H band. The PVDF also were denoted by the peaks at wavenumbers 1403, 1180, 879, and 841 cm^−1^ assigned to C-H vibration, C=C band, asymmetric CCC, and vibration of C-F stretch, respectively [34,35]. New absorption peaks can also be observed at 1525 and 1637 cm^−1^ on the modified membranes (M_2_, M_3,_ and M_4_) associated with C=O stretching vibrations originating from the polyphenol group in TA. Overall, the FTIR spectra could detect the presence of residual TA in the membrane matric, together with surface morphology, and affect the membrane surface hydrophilicity and membrane fouling propensity as shown from the FRR data (Section 3.2.3).

#### 3.1.3. Porosity, Pore Size, and Water Uptake

**Figure 3:**
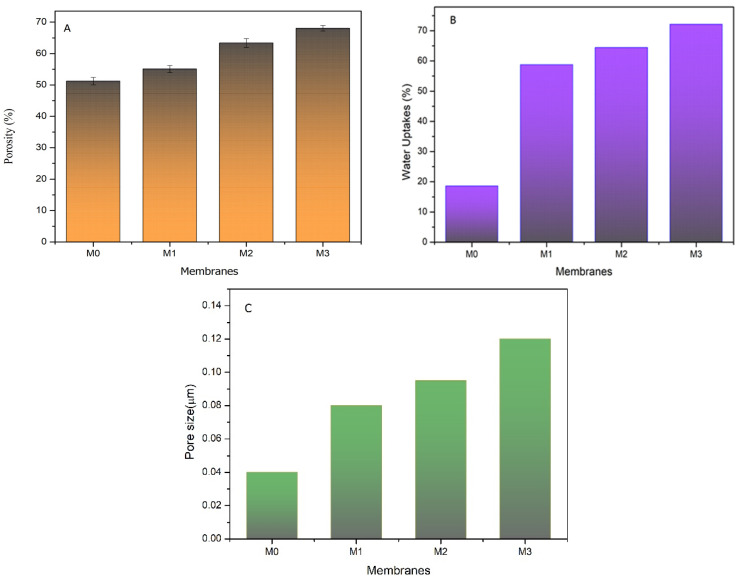
Porosity (**A**), water uptake (**B**), and pore size (**C**) of the developed pristine PVDF membrane (M_0_), and with the addition of tannic acid at 1 (M_1_), 1.5 (M_2_), and 2 wt% (M_3_) in the dope solutions.

Figure 3 shows the porosity, water uptake, and pore size of the prepared membranes. They are considered important membrane characteristics that also affect filtration performance. Based on Figure 3A, increasing the dosage of TA led to higher membrane porosity. The porosity of the membrane slightly increased from 51.22% to 55.08%, 63.39%, and 68.08% for M_0_, M_1_, M_2_, and M_3_, respectively. The enhanced porosity under higher TA loadings was caused by the increasing amount of polyphenol in TA [16]. The hydrophilic TA migrated toward the water phase during the phase inversion leaving the void space behind. The hydrophilic nature of the TA also destabilized the dope solution allowing faster migration of the water phase into the dope solution phase during the phase separation. Water transport to the membrane matric formed polymer lean-phase that eventually become the voids that contributed to higher bulk porosity.

Figure 3B shows that the water uptake value was significantly higher for TA-contained membrane, with increasing values for higher TA loadings. Water uptake was defined as the quantity of water occupied by the membrane pore. It was affected by both the surface hydrophilicity and the bulk porosity, being higher for highly porous and more hydrophilic membranes. There was no indication of swelling that otherwise would lower the water uptake, suggesting the small amount of hydrophilic fraction (of TA) in the polymer matric. Residual TA in the membrane matric was likely located near the pore wall due to its likelihood of joining the polymer lean-phase during the phase inversion. As discussed later, suitable wettability, porosity, and water uptake were expected to enhance the permeability.

Figure 3C shows the positive correlation between TA dosing with the resulting membrane pore size estimated using Equation (3). Enlargement of the membrane pore size was proven by the decrease in HA rejection, as discussed later. The porosity and pore size trends agreed well with an expected instantaneous demixing during the phase inversion. Hydrophilic HA in the dope solution makes it less stable thermodynamically. The rate of solvent/nonsolvent exchange during the phase inversion increased resulted in a faster demixing process, more porous structures, and larger pore sizes. The active substance in the TA additive also diffuses into the nonsolvent solution because of its suitable affinity to water, allowing the formation of pores with larger size [12].

#### 3.1.4. Membrane Morphology

**Figure 4:**
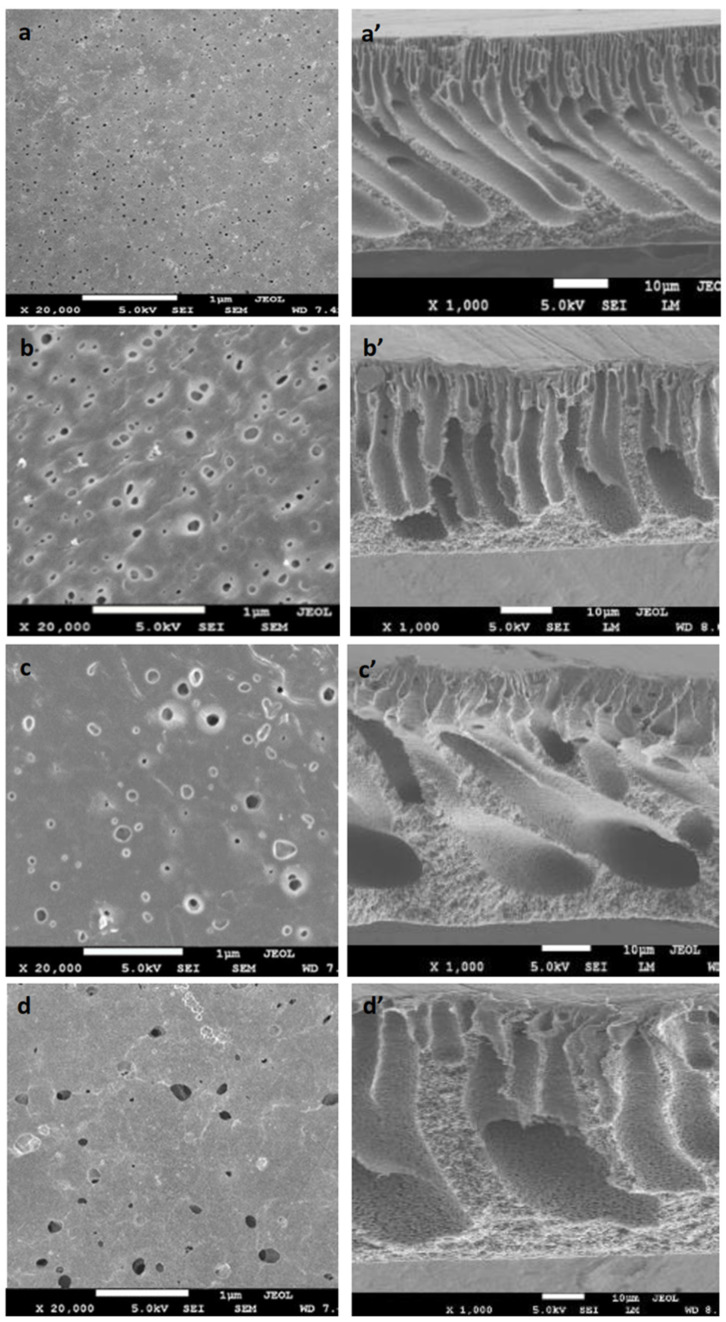
SEM images of the prepared membranes: (**a**) M_0_ surface, (**a’**) M_0_ cross-section; (**b**) M_1_ surface, (**b’**) M_1_ cross-section; (**c**) M_2_ surface, (**c’**) M_2_ cross-section; (**d**) M_3_ surface, (**d’**) M_3_ cross-section.

Figure 4a–d shows the surface view of the prepared membranes. The pristine PVDF membrane (M_0_) had a smooth surface with a homogeneous spatial distribution of surface pores (black dots). The addition of TA significantly altered the number and the size of the surface pores. Pores of the TA-modified PVDF membranes (M_1_, M_2_, and M_3_) were larger than the pristine PVDF (M_0_).

Figure 4a’–d’ shows the cross-section image of each membrane. All prepared membranes exhibited an asymmetric structure consisting of two layers: the dense top layer and the porous bottom layer, a typical phase-inverted membranes structure. It can be observed that higher TA loadings in the dope solutions caused the resulting membranes to be more porous and had larger pore sizes. The findings are consistent with the data in Figure 3A,C. Additive TA is hydrophilic due to the polyphenol group it contains. When blended in the membrane system, the hydrophilic particles had strong interactions with the nonsolvent. It led to the instantaneous demixing during the phase separation resulted in the formation of polymer matric with a more porous structure and larger pore sizes [12]. The findings in Section 3.1.1, Section 3.1.2, Section 3.1.3 and Section 3.1.4 demonstrated that blending of TA into the dope solution offered dual advantages of improving the surface chemistry by lowering the water contact angle and providing polar groups on the membrane surface to guard adhesion of foulant, as well as improving the structural parameters (pore properties) that beneficial to lower the intrinsic membrane resistance. Nonetheless, the surface water contact angles of the developed membranes were still higher than most membranes developed via the application of TA as the surface coating agent summarized elsewhere [14].

### 3.2. Membrane Filtration Performance

#### 3.2.1. Pure Water Flux

**Figure 5:**
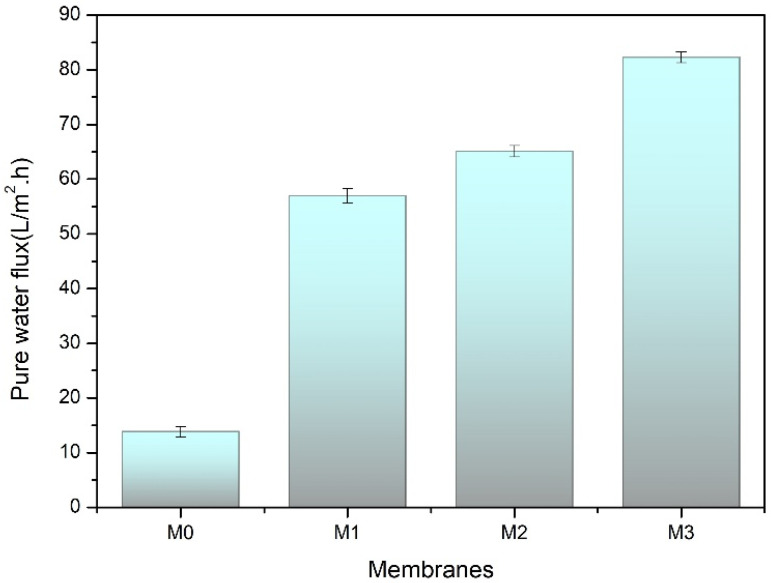
Pure water flux of the developed pristine PVDF membrane (M_0_), and with the addition of tannic acid at 1 (M_1_), 1.5 (M_2_), and 2 wt% (M_3_) in the dope solutions.

Figure 5 displays the pure water flux of the PVDF membranes without and with TA as an additive. That pure water permeation increased with the increasing concentration of TA in the dope solutions. At operating pressure of 1 bar, pristine PVDF membrane (M_0_) produces a flux of 15.04 L/m^2^.h, whereas the fluxes of the TA-modified membranes were 20.7, 35.6, and 50.9 L/m^2^.h for M_1_, M_2_, and M_3_, respectively. This increase in flux is brought by the improved membrane pore properties after modification with TA, as confirmed by the membrane morphology from FE-SEM analysis (Figure 4).

#### 3.2.2. Humic Acid Solution Permeation and Rejection

**Figure 6:**
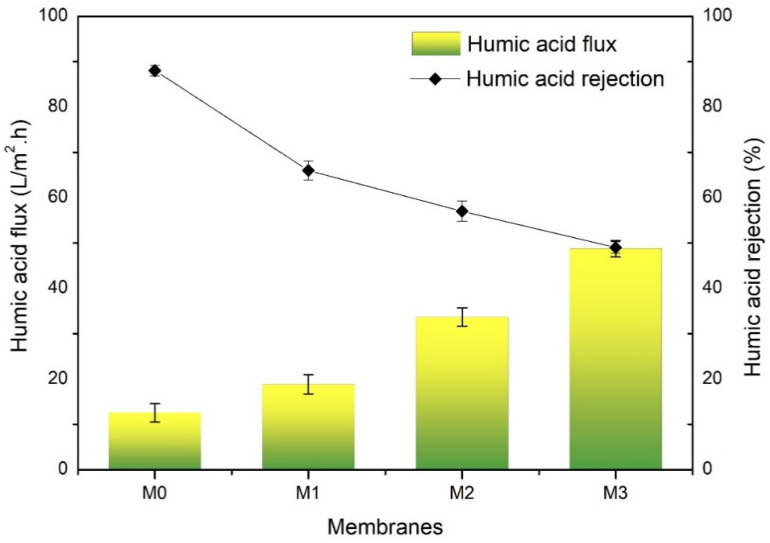
Humic acid flux and rejection of the developed pristine PVDF membrane (M_0_), and with the addition of tannic acid at 1 (M_1_), 1.5 (M_2_), and 2 wt% (M_3_) in the dope solutions.

Figure 6 shows the relation between HA solution flux and its rejection of the pristine PVDF and TA-modified PVDF membranes. It shows that an increase in the flux of HA solution is seen for TA-modified membranes (M_1_, M_2_, M_3_), which is due to the increase in porosity and pore size. The ability of a membrane to separate the contaminant particles in water is essential in membrane separation. Membrane with high water flux and a high percentage of rejection value is preferred in membrane filtration. However, in reality, water flux and solute rejection tends to be inversely proportional and is often closely related to the membrane pore properties. Commonly, a membrane with a larger pore size generates higher flux. However, the large pore size allowed more solute to permeate, which reflects on the lower rejection.

In terms of HA rejection performance, the pristine PVDF (M_0_) membrane had the highest rejection (96.5%) because of its dense structure. The addition of TA into the membrane successfully forms larger pores on the membrane surface, thus decreasing the HA rejection. The decrease in rejection is because some HA particles managed to pass through the membrane pores [10]. It is worth noting that the poor rejection here does not imply a poor performance in the actual applications. The HA solution was selected as the fouling prone feed because it presents in the surface water (i.e., river water), commonly called natural organic matter. Its presence in surface water ranges from a few ppm and is typically removed through the coagulation/flocculation process in combination with membrane filtration. The more important aspect of the filtration performance is the ability of the membrane to maintain its filtration flux by resisting the fouling from HA in the feed solution, as discussed in Section 3.2.3.

#### 3.2.3. Antifouling Performance

**Figure 7:**
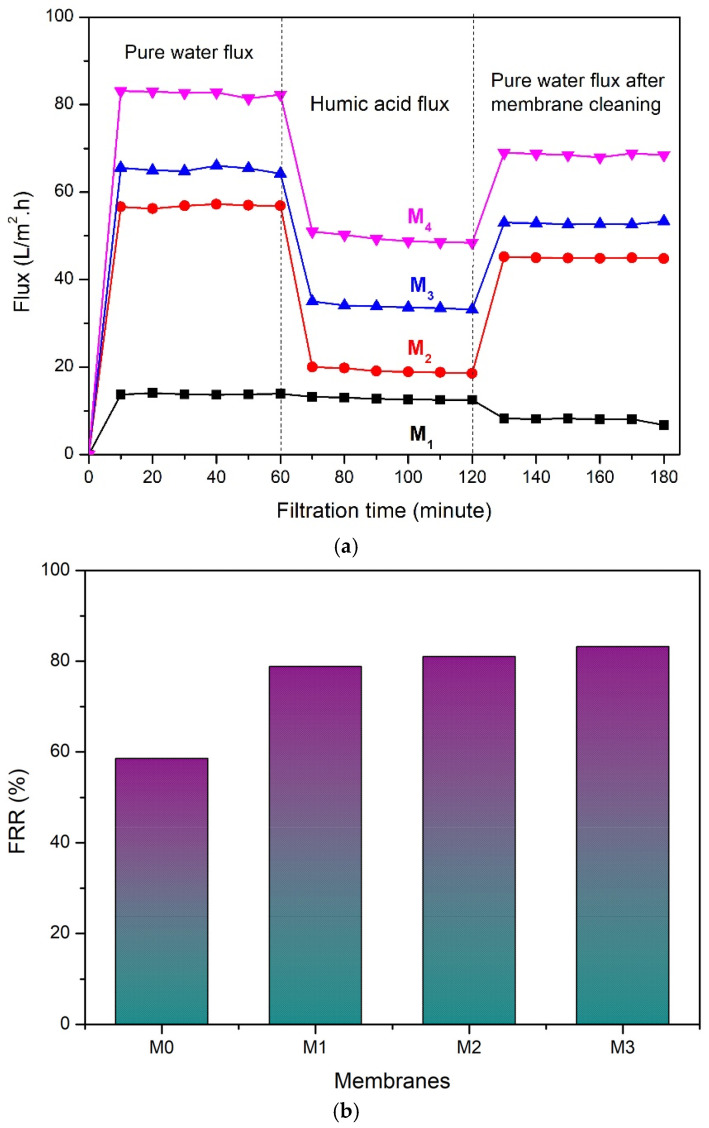
(**a**) Water flux profile of membranes during filtration process; (**b**) FRR values of the developed pristine PVDF membrane (M_0_), and with the addition of tannic acid at 1 (M_1_), 1.5 (M_2_), and 2 wt% (M_3_) in the dope solutions.

Figure 7 shows the FRR result for all prepared membranes demonstrating the advantage of TA-modified PVDF membranes that posed significantly higher FRR values. FRR is typically used as an indicator of membrane capability in recovering the flux performance after membrane backwashing [36]. The FRR also indicates the fouling resistance of the membrane by observing how easy the fouled membrane can be backwashed [13].

Figure 7a displays the profile of flux loss that occurred during the filtration experiment. As seen, the initial pure water flux is high due to no occurrence of fouling in the earlier stage of the filtration; however, when the feed was changed to HA, the flux declined almost half the portion of the pure water flux due to the presence of HA particles in the feed that increases the permeation resistance and the flux reduced further due to accumulation of the HA particle on the membrane surface that is widely known as fouling. Hereafter, the membrane was cleaned by means of backwash, and a pure water flux experiment was conducted once again. The pure water flux after cleaning is much lower compared to the initial pure water flux because of flux loss that occurred due to the fouling. These data are used to evaluate the antifouling performance of the membrane in the form of FRR, which is given in Figure 7b. Figure 7b shows that M_0_ had the lowest FRR-value suggesting that it suffered most of the flux loss caused by membrane fouling. The pristine PVDF (M_0_) membrane had an FRR value of 58.54%, implying a small ratio of the original flux that can be successfully restored. The backwashing failed to recover the performance as the fouled membrane was not easily cleaned. It can be attributed to the hydrophobic nature of PVDF that facilitates strong membrane-foulant interactions [37]. Meanwhile, the FRR value for the TA-modified PVDF membranes increased with the increase in the concentration of TA in the dope solution. The addition of TA at concentrations of 1%, 1.5%, and 2% (on M_1_, M_2_, M_3_) increased the FRR values to 78.34%, 81%, and 83.17%, respectively. The increase in the FRR is because the membranes had improved hydrophilicity with the addition of TA. The hydrophilic nature of the membrane resulted in poor interaction with hydrophobic foulant. This way, the fouled membrane can be washed off easily via a simple backwash.

### 3.3. Membrane Stability

**Figure 8:**
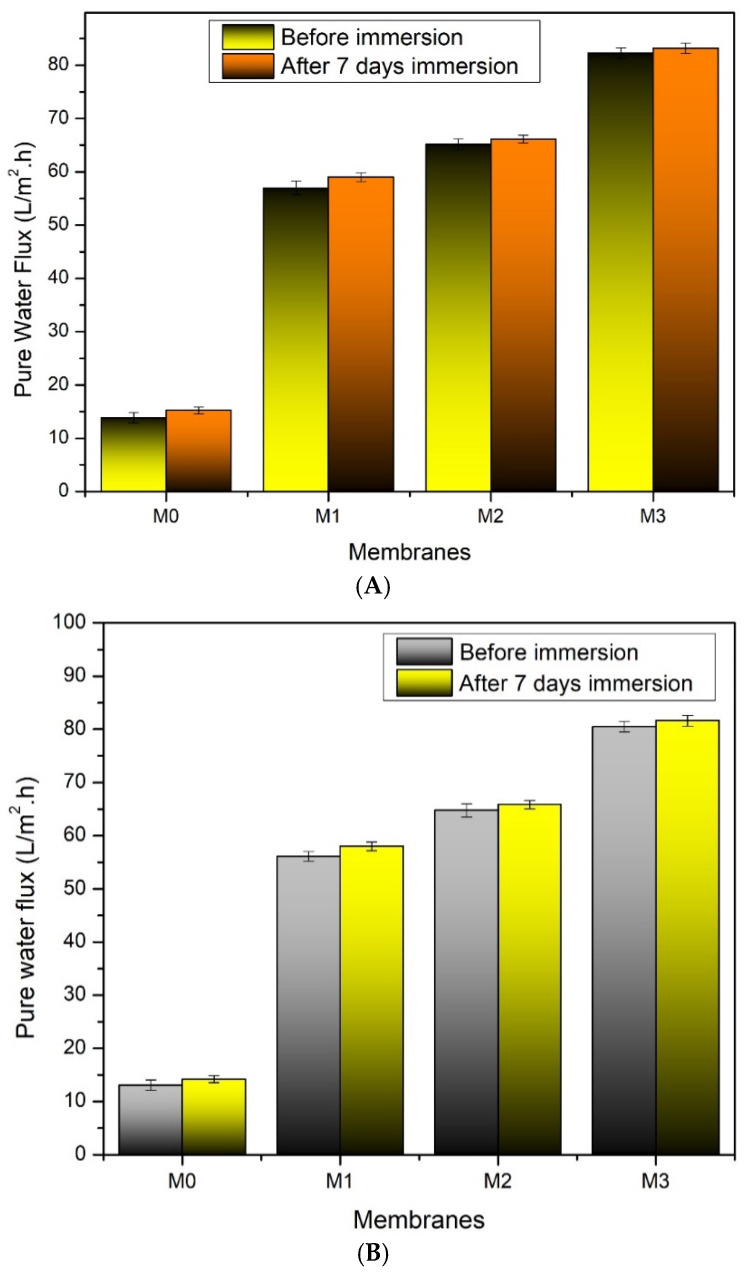
Pure water flux of the developed pristine PVDF membrane (M_0_), and with the addition of tannic acid at 1 (M_1_), 1.5 (M_2_), and 2 wt% (M_3_) in the dope solutions before and after immersion in (**A**) HCl 1N (pH = 1), and (**B**) NaOH 1M (pH = 14) for 7 days.

Figure 8 shows the pure water flux for all PVDF membranes before and after immersion in acid (pH 1) or alkaline (pH 14) solutions. A membrane stability test was conducted to investigate the stability of TA as the additive when the TA-modified membrane is exposed to harsh conditions during actual applications (i.e., acid or base cleanings). The test was performed by exposing the membranes to strong acid and alkaline solutions for a week, then the water flux before and after a certain duration of exposure was compared. Overall findings suggest the suitable stability of the membranes under harsh conditions. Slight increments of the water fluxes were observed in all cases but were statistically insignificant. Judging from the high stability of PVDF, a small change in flux can be attributed to the degradation of residual TA that left the void and turned the membrane structure to be more porous and/or the membrane pore to be larger, as also occurred when employing a dragon blood in resin [6], PEG [38] or PVP as additive [39]. However, the water flux increment for the pristine PVDF membranes suggests that the treatment might also affect the PVDF polymer. The cause of the slight instability issue at this moment is still unclear and could not be explained from the available data and is worthy of further investigation.

## 4. Conclusions

This research explores the performance of TA as a modifying agent for the preparation of PVDF UF membranes. It was found that the addition of TA enhanced the resulting membrane properties, both in terms of surface chemistry (hydrophilicity) and physical structure. The incorporation of TA as an additive increased the bulk porosity, water uptake, and water contact angle of the resulting membranes. Those properties translated into improved clean water flux from 15.4 L/m^2^.h for the pristine PVDF membrane up to 50.9 L/m^2^.h for the TA-modified membranes with 2 wt% loading. The antifouling performances of the TA-modified membranes were significantly higher than the pristine PVDF membrane (FRR = 58.54% vs. FRR 78–83%). The TA-modified membranes were also stable under exposure to harsh acid and harsh alkali conditions. The positive results were due to the hydrophilicity enhancement of the membrane thanks to the presence of TA residue within the polymeric membrane matric, which is rich in hydrophilic polyphenol groups. However, the larger pore size of the TA-contained membranes lowered increased pore size and brought about the decrease in HA rejection. Overall, employing TA as a dope solution additive resulted in enhanced performance of the resulting phase PVDF-based UF membranes. This finding provides a straightforward method for PVDF membrane fabrication to enhance membrane properties via the simple blending of cost-effective TA into the dope solution.

## Figures and Tables

**Table 1:** The composition of the dope solution for membrane preparation.

Membrane	PVDF (wt%)	Tannic Acid (wt%)	DMAc (wt%)
M_0_	15	0	85
M_1_	15	1	84
M_2_	15	1.5	83.5
M_3_	15	2	83

## Data Availability

The data presented in this study are available on request from the corresponding author.
